# Our Contemporaries

**Published:** 1914-01

**Authors:** 


### OUR CONTEMPORARIES.

Mr. Abraham Flexner of the Carnegie Foundation, in the *Atlantic* for November, has an interesting article on "The German Side of Medical Education" which every medical man should read—partly because there is a good deal to be said on the other side. "On the minimum standard on which a medical school can live in Germany, over three-fourths of the medical schools of the United States and Canada would be stamped out of existence." Last August, 22 of 106 United States medical schools were rated as A plus, several schools have since been closed and several give only partial courses, leaving about 95 full time schools not destined to speedy extinction. The Canadian schools number nine, most of them being high grade. If "A plus" includes schools which Germany would not even tolerate, our classification ought to be amended. There are 33 Class A schools which are considered as satisfactory in preliminary requirements, length and scope of curriculum, but lacking in certain details, mainly on account of money. It occurs to us that Mr. Flexner's statement should be officially investigated, for the two divisions of Class A comprise more than 50 per cent. of all the schools in this country, and we dislike to think that all of the ordinary Class A schools and some of the Class A plus would be closed according to German standards.

Mr. Flexner states that New York is the only state in which there is a Department of Education having the power to maintain a decent minimum, and that "in the city and state of New York, medical schools still exist which are utterly incapable of fulfilling respectably the purpose for which they purport to have been established." Considering that eight or ten states and nearly three-fourths of the medical colleges have established matriculation standards higher than those of New York state, it is in no spirit of discourteous contradiction that we question whether New York is the only state having such powers of control and, considering that Columbia is mentioned in such a way as to imply that it is not meant and that there are only nine other medical schools in New York state, three outside of New York city and six in the city that confer degrees, his censure is just close enough to personalities to warrant a demand for names and an opportunity for argument. For example, if someone announces publicly that Mr. Jones is not fit to live, the distrust aroused and the embarrassment of Mr. Jones are not nearly so great as when attention is called to a group of three men of which Mr. Jones is one, and the equivocal statement is made that one of these three men is not fit to live, nor is any injustice done to the other members of the group. But Mr. Flexner's remark is made in such terms that, while he calls attention to both of these groups of three and six colleges, he does not even limit his assertion to one of each but places a stigma on all by his remark, and puts any one of them that may feel inclined to defend itself in the light of a confessed culprit.

When we realize that approximately 75 per cent. of all medical schools of the United States have adopted a standard of matriculation including at least one year of collegiate work, and that these schools include almost every one that makes any serious contention of being in the running for a permanent and respectable position, it seems that we may fairly claim equality with the "gymnasium" requirement of Germany. The gymnasium student undoubtedly works harder and enters more into detail than the high school and freshman college student, but the general educational scope is not widely different, and it is open to debate whether the intellectual maturity and preparedness for technical instruction in medicine, as well as ambition to do good work before and after graduating in medicine, is not equally great in the American student. Thirty-two medical schools of the United States require two years or more of collegiate study for matriculation. Unless these are weak in other respects, they surpass by a considerable degree Mr. Flexner's allowance of something less than 25 per cent. that do not come up to the German standards.

Mr. Flexner's plea for an elective course in medical schools seems to us untenable under existing circumstances, except to the degree to which it has long been practiced in presenting subsidiary lectures which the student was not absolutely required to attend, or in allowing students of special proclivities to take up work as prosectors, laboratory assistants, etc., certain advanced and recondite parts of ordinary courses not required of every one. The analogy to baccalaureat elective courses does not seem to us well chosen. In very few if in any American colleges is the student allowed to make promiscuous choice of studies. He is not only required to follow with reasonable thoroughness any single course selected, but he is usually required to group his courses so as to form a reasonably unified and consistent whole. On completing his scholastic education, he is again allowed to choose, among medicine, law, theology or a number of other courses fitting for a life work. Having made this choice, further election, beyond a very minor degree, is impossible under existing conditions. The medical school does not purport to graduate specialists; in fact the majority view of those best fitted to judge is that specialism should not only follow and not supersede the ordinary medical course, but that it should follow some years of experience in general practice or, at least, of extended post graduate study. With the minor exceptions already noted, there is not a study in the medical curriculum that can be omitted without jeopardizing the interests of the medical student and of his prospective patients—for even Mr. Flexner must recognize that the majority of medical students are such on account of the intention of actually practicing medicine, however infra dig. such an ambition may be, and there are even medico-legal complications that might arise if the medical schools allowed much option in the choice of studies.

---

The *Medical Fortnightly* of St. Louis, Nov. 25, 1913, reproduces our editorial on Independent Medical Journals and Official Organs.

---

The *International Hospital Record* perpetrates the epigram "Surgically top-heavy hospitals," which is worth thinking about.

---

Dr. Charles E. Woodruff of New Rochelle, N. Y., has been appointed co-editor of *American Medicine* and will have charge of the Abstract Department.

---

The *Annals of Surgery* for December is almost double its usual size, being a special Anaesthesia number, but it contains a number of valuable articles in addition to this symposium.

---

The *Illinois Medical Journal,* Dec., 1913, speaks rather unfavorably of the American College of Surgeons and of certain phases of the A. M. A. franchise. It is the official organ of the State Society.

